# Evolving Paradigms in Chronic Graft-Versus-Host Disease: From Global Immunosuppression to Precision Targeted and Cell-Based Therapies

**DOI:** 10.1007/s12015-026-11131-7

**Published:** 2026-04-20

**Authors:** Yu Wang, Wanying Liu, Xiaobing Huang, Yi Xiao

**Affiliations:** 1 Department of Hematology, Tongji Hospital, Tongji Medical College, Huazhong University of Science and Technology, No. 1095, Jiefang Road, Qiaokou District, Wuhan, Hubei 430030 P.R. China; 2 Department of Hematology, Sichuan Academy of Medical Sciences & Sichuan Provincial People’s Hospital, No. 32, West Section 2, First Ring Road, Chengdu City, Sichuan P.R. China 611072

**Keywords:** Chronic graft-versus-host disease, BTK inhibitors, ROCK2 inhibitors, JAK inhibitors, Anti-CSF-1R monoclonal antibodies, Cellular immunity, Mesenchymal stem cells

## Abstract

Allogeneic haematopoietic stem cell transplantation (allo-HSCT) is a well-established treatment for a range of medical conditions. However, the procedure carries with it a significant risk of chronic graft-versus-host disease (cGVHD), which can have a considerable impact on patients’ quality of life and clinical outcomes. In recent years, there has been a marked shift in treatment strategies for this condition, with greater emphasis being placed on novel targeted therapies and cell therapies that target specific pathways. This shift in approach signifies a more profound comprehension of the pathogenesis of the condition, particularly with respect to abnormal T/B cell activation, tissue fibrosis progression, and immune tolerance imbalance. This article systematically reviews the current treatment framework and latest advances in cGVHD, covering the evolution from first-line immunosuppression to diverse precision targeted drugs and cellular therapy strategies. The study methodically analyses their positioning in clinical practice and the challenges encountered, with the objective of providing theoretical support and practical guidance for refined, individualised clinical management of cGVHD.

## Foreword

Allogeneic haematopoietic stem cell transplantation (allo-HSCT) remains a pivotal curative approach for various haematological malignancies, certain inherited metabolic disorders, and primary immunodeficiency syndromes. The efficacy of this treatment is contingent upon graft-mediated immune activity, particularly the graft’s capacity to clear leukemia or tumour cells. However, when this immune response becomes overactivated or loses specificity, it abnormally attacks the recipient’s normal tissues and organs, thereby inducing graft-versus-host disease (GVHD). The classification of GVHD is typically based on the timing of onset, clinical-pathological characteristics, and immunological mechanisms. The two main types of GVHD are acute (aGVHD) and chronic (cGVHD). Among these, cGVHD is the most prevalent and complex mid-to-long-term complication post-transplantation, becoming a critical factor limiting patients’ long-term quality of life and overall prognosis. From a clinical perspective, cGVHD manifests as a highly heterogeneous condition, fundamentally characterised by systemic immune dysregulation and the development of fibrosis [1]. Glucocorticoids, when administered systemically, have been established as the prevailing initial treatment for cGVHD. However, it is important to note that long-term steroid use can lead to a range of complications [2, 3]. Furthermore, approximately 40%–50% of patients exhibit poor response to glucocorticoids or experience disease recurrence during dose tapering, progressing to steroid-resistant cGVHD [4]. These patients generally have a poor prognosis, presenting a significant challenge in clinical management. In recent years, researchers have deepened their exploration of cGVHD pathogenesis, particularly in core pathways such as abnormal T/B cell activation, the immune-fibrosis network, immune tolerance imbalance, and microenvironment remodeling. Consequently, treatment strategies for cGVHD have gradually shifted from traditional non-specific immunosuppression towards novel targeted interventions focused on key pathways. For instance, small-molecule inhibitors targeting the JAK/STAT signaling pathway have been shown to modulate immune responses mediated by multiple inflammatory cytokines; ROCK2 inhibitors have been demonstrated to influence fibrosis progression by regulating cytoskeletal organization and immune cell migration; BTK inhibitors function by blocking B-cell receptor signaling and antigen presentation; and interventions targeting the TGF-β pathway are also under exploration to reverse tissue fibrosis. Furthermore, a range of cell therapies, including regulatory T-cell infusion and mesenchymal stem cell(MSC) applications, present innovative approaches for restoring immune tolerance and tissue repair. The present article aims to systematically review existing strategies and recent advances in cGVHD treatment, further explore the guiding significance of real-world data for clinical decision-making, and conduct an integrated analysis of current theories and practices. The objective of this study is to establish academic foundations and developmental directions for the refined assessment, individualised treatment, and long-term management of cGVHD.

## Overview of cGVHD

cGVHD is defined as a clinical pathological syndrome that occurs in patients more than 100 days following allo-HSCT. During the process of reconstitution of the immune system of the recipient by the donor, immune cells derived from the donor recognise the host’s tissue antigens as “non-self”, thereby initiating an immune attack that leads to organ damage in the recipient. cGVHD can involve multiple organ systems, including the skin, oral cavity, eyes, lungs, liver, muscles, fascia, and joints, and can cause pain, functional impairment, and activity limitations [5]. For instance, cutaneous involvement may result in pruritus, sclerotic changes ranging from superficial lichen sclerosus-like features to deep sclerotic features with hidebound skin, or ulceration. Sclerotic features encompass a spectrum of manifestations: deep sclerotic features involve the reticular dermis and/or subcutaneous tissue causing joint contractures, morphea-like features present as localized sclerotic plaques, and lichen sclerosus-like features represent superficial papillary dermal involvement with white atrophic patches [6].

**Fig. 1:**
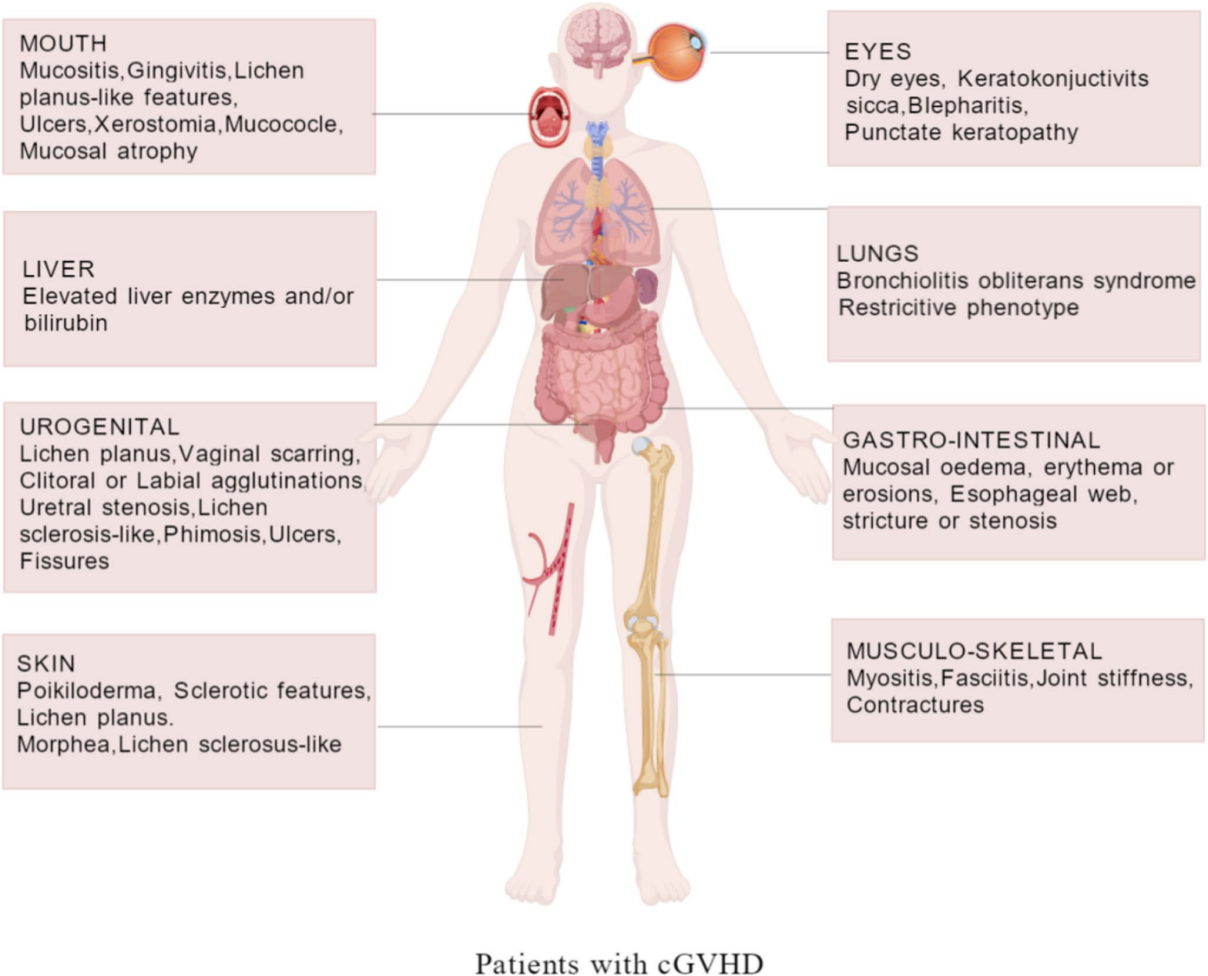
Clinical manifestations associated with various organ

The manifestation of oral cGVHD is frequently characterised by a triad of symptoms: oral pain, dryness, and sensitivity. These symptoms have the capacity to result in a substantial impairment of patients’ dietary and speech functions [7]. Furthermore, it has been demonstrated that pulmonary involvement may result in bronchiolitis obliterans syndrome (BOS), which is characterised by dyspnoea and subsequent restriction of patients’ mobility [8–10]. Additionally, a restrictive phenotype with reduced total lung capacity, representing an atypical manifestation of pulmonary cGVHD, may occur. It has been demonstrated that renal involvement may even progress to nephrotic syndrome [11]. According to the 2014 NIH consensus criteria, cGVHD manifestations are classified into diagnostic features (e.g., poikiloderma, lichen planus-like features, sclerotic features, lichen sclerosus-like features, esophageal web, sicca syndrome), distinctive manifestations requiring confirmation (e.g., depigmentation, fasciitis), other non-specific features, and common manifestations shared with acute GVHD [12]. Clinical manifestations associated with various organ involvement are shown in Fig. 1.

The incidence of cGVHD varies across different study populations but remains generally high. As demonstrated, the 2-year cumulative incidence of cGVHD is estimated to be approximately 39% [13]. Furthermore, the incidence of cGVHD is closely associated with transplant type, GVHD prophylaxis strategies, donor and patient age, and patient baseline characteristics. Advanced donor age and older patient age are established risk factors for increased cGVHD incidence and severity. A large CIBMTR study demonstrated that each decade increase in patient age is associated with a 13% increased risk of cGVHD (HR 1.13, 95% CI 1.07–1.19, *P* < 0.001), and each decade increase in donor age with a 9% increased risk (HR 1.09, 95% CI 1.03–1.16, *P* = 0.002) [14]. Pediatric studies further confirm that adolescents (13–18 years) have significantly higher cGVHD risk compared to younger children (2–12 years; HR 0.32, *P* < 0.001), and that using a younger donor significantly reduces cGVHD risk (HR 0.43, *P* = 0.0014) [15]. Additionally, the incorporation of rabbit anti-thymocyte globulin (rATG) into GVHD prophylaxis has been demonstrated to significantly reduce cGVHD incidence. A landmark phase III randomized trial showed that rATG reduced cGVHD from 69% to 32% (*P* < 0.001) in matched unrelated donor transplantation, resulting in superior chronic GVHD-free, relapse-free survival (37% vs. 17%) [16]. Similar benefits have been observed in matched sibling donor peripheral blood stem cell transplantation, where rATG reduced 2-year cGVHD incidence from 61.4% to 19.3% (*P* < 0.001) [17]. The protective effect of ATG has also been confirmed in reduced-intensity conditioning regimens [18], supporting its use across various transplant platforms. Research has demonstrated that patients undergoing post-transplant cyclophosphamide (PTCY) for the prophylaxis of cGVHD exhibit a significantly reduced two-year incidence of moderate-to-severe cGVHD when compared to those undergoing tacrolimus-based regimens (12% vs. 36%) [19]. As demonstrated in, patients undergoing peripheral blood stem cell transplantation (PBSCT) exhibit a significantly higher incidence of cGVHD compared to those receiving bone marrow transplantation (BMT) [20]. The incidence of cGVHD is higher in unrelated donor (URD) transplants, particularly in URD mismatched patients. Research has indicated that the 2-year cGVHD rate is 51% for URD mismatched patients, in comparison to 13% for haploidentical patients [21]. Furthermore, the degree of donor-recipient gender matching has been demonstrated to influence the incidence of cGVHD. For instance, male recipients who undergo transplantation from female donors are subject to a higher risk of cGVHD [22]. The National Institutes of Health (NIH) consensus statements from 2005 to 2014 provided a framework for the standardisation of the diagnosis and grading of cGVHD, emphasising the integration of clinical manifestations and histological features, with the grading of affected organs based on the number and severity of the manifestations present [1, 5, 6]. For instance, the occurrence of skin ulceration is defined as the most severe manifestation of cutaneous cGVHD involvement [23, 24]. Pulmonary tissue impairment may manifest as a decrease in lung capacity during activities requiring minimal exertion, accompanied by a forced expiratory volume in one second (FEV1) percentage range of ≤ 39% or 40%−59% with significant clinical symptoms. According to the NIH consensus criteria, severe pulmonary cGVHD is defined by FEV1 ≤ 39% or the requirement of supplemental oxygen, while FEV1 of 40%−59% typically represents moderate involvement [25, 26].

## Immunological Mechanisms of cGVHD

**Fig. 2:**
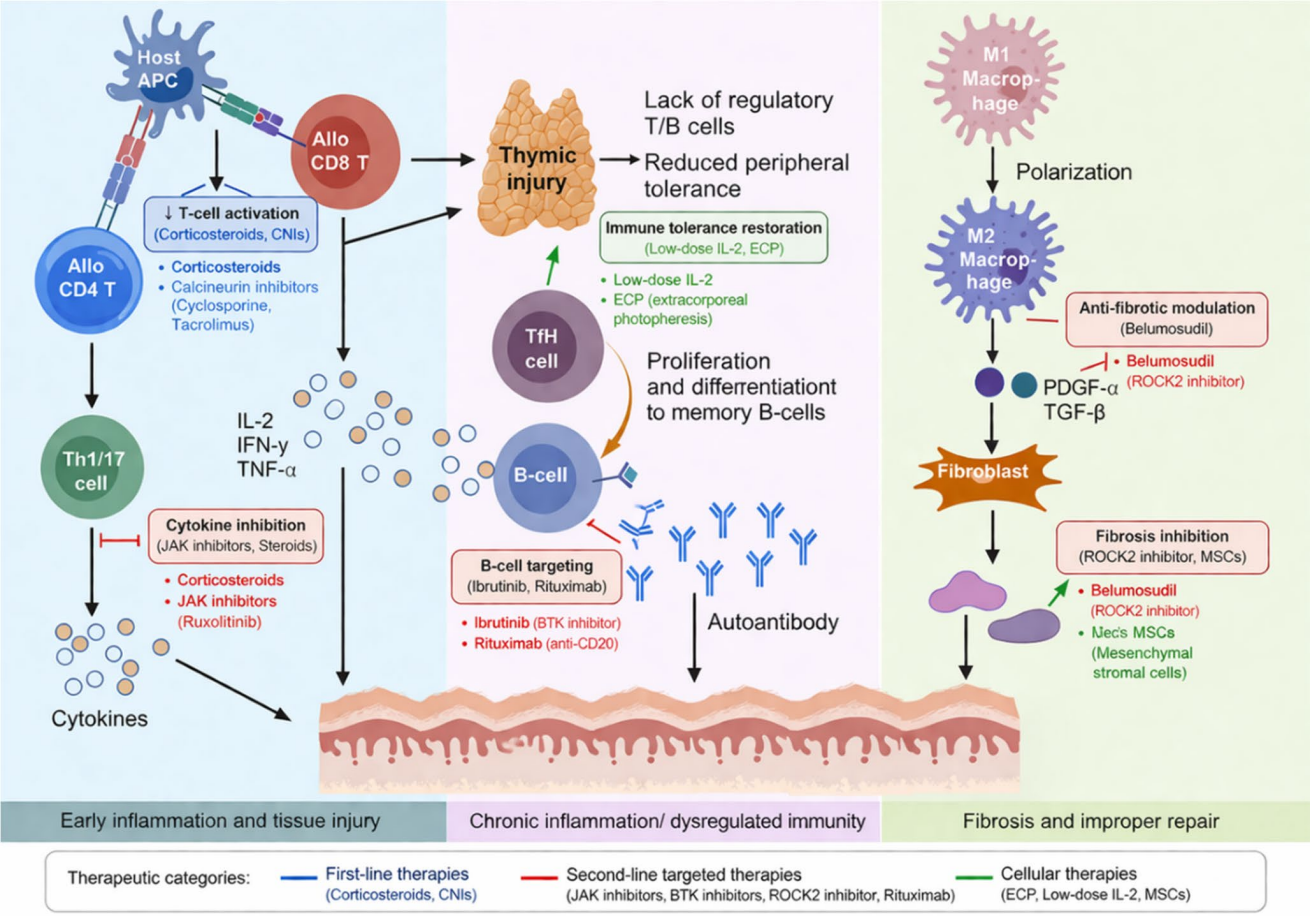
Immunopathogenesis of cGVHD and therapeutic targets of current treatment strategies. cGVHD arises from donor T-cell activation by host antigen-presenting cells, leading to Th1/Th17 polarization and proinflammatory cytokine production (IL-2, IFN-γ, TNF-α). Corticosteroids and calcineurin inhibitors suppress T-cell activation, while JAK inhibitors attenuate cytokine signaling. Thymic injury and impaired immune tolerance promote TFH-driven B-cell activation and autoantibody production, which can be targeted by BTK inhibitors (ibrutinib) and anti-CD20 antibodies (rituximab). In the fibrotic phase, M2 macrophages drive TGF-β– and PDGF-mediated fibroblast activation and extracellular matrix deposition. ROCK2 inhibition (belumosudil) modulates both immune dysregulation and fibrosis, while cellular therapies (ECP, low-dose IL-2, MSCs) restore immune tolerance. Color coding: blue, first-line therapies; red, targeted agents; green, cellular therapies

The mechanism of cGVHD is centred on multi-organ inflammation and fibrosis [27]. The NIH have proposed a three-phase model for cGVHD. The first phase is an early acute inflammatory phase, which is characterised by acute inflammatory responses. The second phase is a chronic inflammatory and dysimmunological phase, which is manifested by sustained immune system activation and dysregulation. The third and final phase is a tissue fibrosis phase, which ultimately leads to organ fibrosis and dysfunction (Fig. 2) [28]. This process involves complex interactions between host antigens and donor immune responses. Initially, donor T cells recognize host major histocompatibility complex (MHC) antigens or minor histocompatibility antigens (mHA) presented by host antigen-presenting cells (APCs), which results in T cell activation(e.g., Th17, Tfh) while concurrently suppressing Treg cell numbers and function [29]. The direct destruction of host cells by these activated T cells is achieved through the release of perforin, granzyme, and Fas/FasL-mediated apoptotic signals [30, 31]. Th17 cells have been shown to release pro-inflammatory cytokines, including IL-2, IL-17 A, IFN-γ, GM-CSF and TNF-α, which in turn exacerbate the inflammatory response [32].

Besides, B cells contribute to cGVHD pathogenesis through multiple mechanisms: (1) production of autoantibodies and alloantibodies directed against host antigens; (2) antigen presentation to donor T cells, promoting their clonal expansion and survival; (3) interaction with T follicular helper (Tfh) cells in germinal centers, leading to aberrant B cell activation and differentiation into plasmablasts; (4) cytokine secretion (Th1 and Th2 cytokines) that regulate T cell populations including Tregs; (5) loss of regulatory B cell function—IL-10-producing regulatory B cells are deficient in number and function in cGVHD patients [33]. Patients with active cGVHD consistently have lower numbers of naive and transitional B cells as well as total B cells. B cell activating factor (BAFF) levels are consistently increased relative to B cell numbers, promoting survival of autoreactive B cell clones [34]. The cells in question have been observed to drive B cells to produce autoantibodies, form immune complexes, and activate M1 macrophages via FcγR. This, in turn, has been shown to promote their conversion to M2 macrophages. The latter secrete elevated levels of TGF-β1 and PDGF-B, thereby establishing a persistent fibrotic microenvironment [35].

In chronic inflammatory environments, TGF-β emerges as a core factor driving fibrosis. TGF-β has been demonstrated to directly upregulate COL1A1 and FN1 by activating the canonical Smad2/3 signaling pathway, thereby inducing fibroblast activation and collagen synthesis. Furthermore, TGF-β activates the non-canonical ROCK2 pathway, which in turn remodels stress fibres via Myosin II and LIMK-cofilin. This process enhances α-SMA expression and transdifferentiates fibroblasts into myofibroblasts [36, 37]. Concurrently, ROCK2 directly phosphorylates Foxp3, thereby inducing its proteasomal degradation and consequently reducing the proportion of Tregs, thus maintaining Th17/Tfh dominance [38, 39]. This process perpetuates a pro-fibrotic environment, thereby providing sustained momentum for chronic inflammation and subsequent fibrosis.

## First-Line Treatment for cGVHD

The fundamental approach to the treatment of cGVHD involves the suppression of T-cell and B-cell activation, the reduction of pro-inflammatory cytokine secretion (including IL-6, TNF-α, IL-17), the inhibition of autoantibody production, and the moderation of organ fibrosis progression [30, 40]. Glucocorticoids, whether administered as monotherapy or in combination with calcineurin inhibitors (e.g. cyclosporine, tacrolimus), are considered the preferred initial treatment for cGVHD according to the latest international consensus. Prednisone is recommended at a dose of 1 mg/kg/day [41, 42]. A multitude of studies have demonstrated that this initial treatment regimen rapidly suppresses lymphoproliferation and alleviates high-grade inflammation in affected organs within a period of 4–6 weeks of cGVHD treatment, achieving a 6-month overall response rate (ORR) of 48.6%–62.5% [4, 43]. However, the sustained remission rate at 12 months without additional immunosuppression is only 15%–33%, suggesting poor long-term control. Importantly, several randomized clinical trials evaluating the addition of other immunosuppressive or immunomodulatory agents to first-line corticosteroid therapy have failed to demonstrate a significant improvement in outcomes. For example, the addition of mycophenolate mofetil (MMF) to initial prednisone therapy did not improve response rates or survival [44], and a randomized trial of sirolimus combined with prednisone similarly did not demonstrate clear superiority over standard approaches [45]. Likewise, studies incorporating agents such as hydroxychloroquine or thalidomide into first-line regimens did not yield consistent clinical benefit [46], highlighting the continued reliance on corticosteroids as the backbone of initial therapy despite efforts to improve efficacy.

Real-world cohort studies further indicate that approximately half of patients exhibit an inadequate response to first-line corticosteroid therapy and, based on their clinical characteristics, are ultimately classified as having either steroid-dependent or steroid-refractory cGVHD(SR-cGVHD). Specifically, steroid-dependence refers to disease flare during the tapering process, whereas steroid-refractoriness denotes a lack of initial response to therapy [4, 47]. Moreover, it has been demonstrated that prolonged exposure to glucocorticoid doses that exceed physiological levels can result in a range of adverse effects, including dyslipidaemia, osteoporosis, and an elevated risk of opportunistic infections [3, 48–50]. Therefore, for patients demonstrating no improvement in organ function after four weeks of standard first-line therapy, or those unable to taper prednisone to less than 1.0 mg/kg/day by week eight, escalation to second-line systemic therapy for cGVHD should be considered promptly [25]. For patients receiving glucocorticoid monotherapy, if disease progression occurs within two weeks of initiation or no improvement is observed at six to eight weeks, glucocorticoid-refractory disease should be suspected, and initiation of second-line cGVHD-directed systemic therapy is warranted.

## Second-Line Therapy for cGVHD

**Fig. 3:**
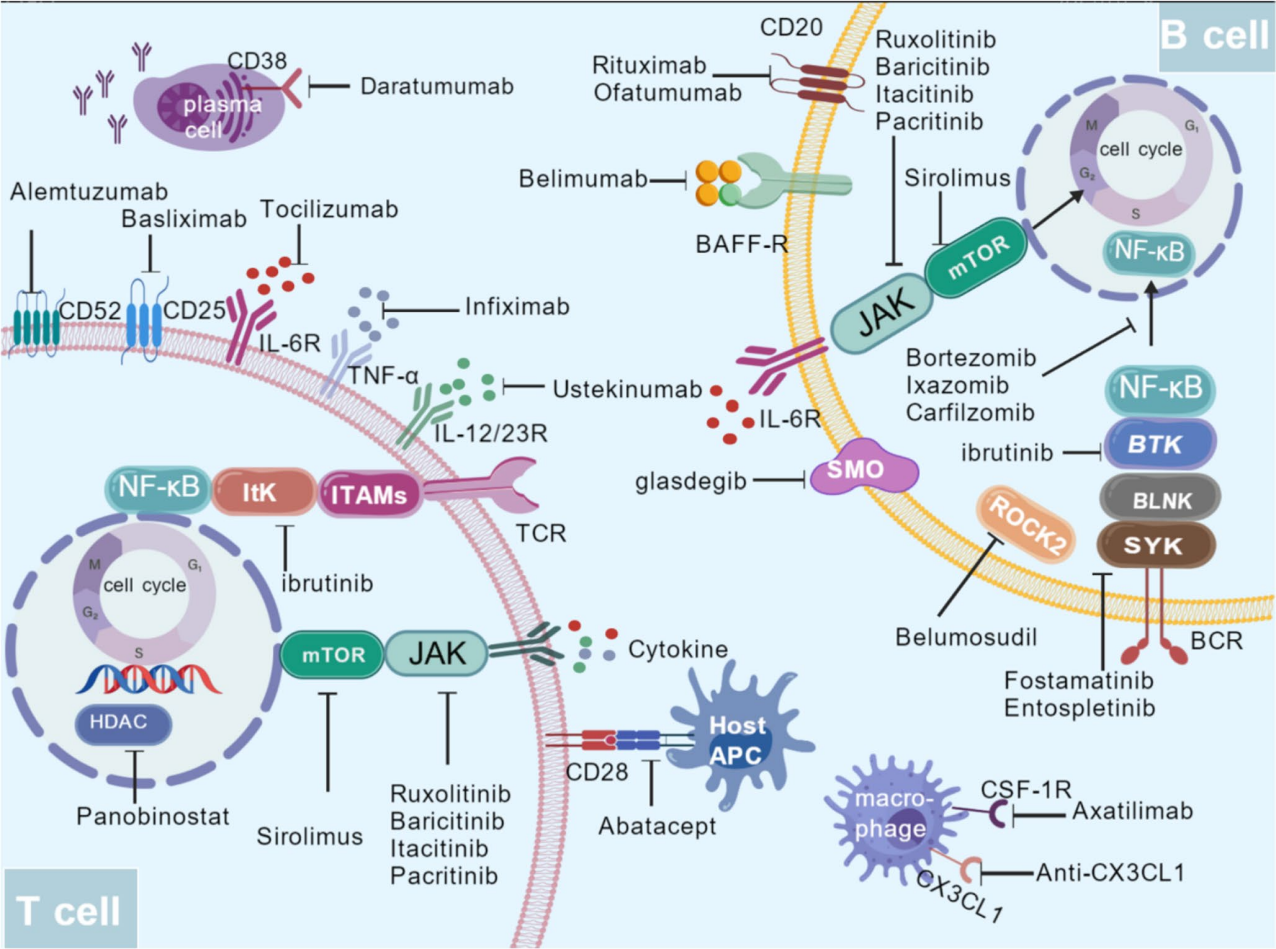
Chronic GvHD development and novel agents targeting B and T cells that are under investigationfor the treatment of the disease. IL-6R, interleukin-6 receptor; TNF-α, Tumor Necrosis Factor-alpha; ITK, 1 L-2-inducible kinase, jAK, janus kinase; mTOR, mammalian target ofrapamycin; HDAC, histonedeacetylase; Tregs, regulatory’T cells; ROCK2, Rho-associated coiled-coil kinase 2; BLNK, B cell linker; NF-kB, nuclearfactor kappa-light-chain-enhancer of activated B cells; ITAMS, immunereceptor tyrosine-based activation motifs; CSF-1R, colony-stimulating factor 1receptor; BCR, B cell receptor; Btk, Bruton tyrosine kinase; Syk, splenic tyrosine kinase; BAFF, Bcell activating factor; BAFF-R, BAFF receptor; CD20, cluster of differentiation 20; CD25, cluster of differentiation 25; CD52, cluster of differentiation52; CD38, cluster of differentiation 38; CD28, cluster of differentiation 28; SMO, Smoothened

To date, a globally unified standard for second-line treatment regimens for cGVHD has not been established. The selection of clinical decisions necessitates a meticulous process, encompassing the evaluation of the affected organs, the severity of the disease, and the individual characteristics of the patient. As of the present moment, a total of three oral medications have been granted regulatory approval for second-line treatment in glucocorticoid-refractory/resistant populations: ibrutinib, Rucaltori and Belumodustil. Axatilimab is also included in this category [51–55]. The present study will examine the three primary pathways of research in this field. The first category comprises conventional immunosuppressants, including methotrexate(MTX) and mycophenolate mofetil. The second category encompasses molecularly targeted agents that target key pathways, such as BTK, JAK, or ROCK2. The third category includes emerging strategies, such as biologics and cellular immunotherapy (Fig. 3). Cross-validation and combined exploration across these directions hold promise for delivering more targeted treatment options for different cGVHD subtypes.

### Traditional Immunosuppressants

#### Methotrexate

MTX is an immunosuppressive agent with distinct applications in both prophylaxis and treatment of graft-versus-host disease [56]. As a cornerstone of GVHD prophylaxis, the combination of calcineurin inhibitors (e.g., cyclosporine or tacrolimus) with MTX has been the standard regimen for preventing both acute and chronic GVHD following allogeneic hematopoietic stem cell transplantation [57]. The mechanism of action involves the competitive inhibition of dihydrofolate reductase (DHFR), thereby blocking the conversion of dihydrofolate to tetrahydrofolate and interfering with DNA and RNA synthesis. This action suppresses rapidly proliferating T cells and B cells and inhibits excessive immune system activation. Furthermore, MTX has been demonstrated to elicit immunomodulatory effects by inducing the proliferation of regulatory T cells (Tregs). MTX also exerts anti-inflammatory effects by inhibiting purine and pyrimidine synthesis, thereby reducing cytokine production [58, 59].

In the treatment setting, MTX is frequently employed as a second-line or steroid-sparing agent for cGVHD. Research indicates that in steroid-refractory cGVHD patients, a 6-month regimen of MTX plus cyclosporine A achieved an ORR of 77.6% (complete response (CR) 59.2%, partial response (PR) 28%) [60]. This suggests that MTX-based therapy may provide meaningful clinical benefit in steroid-refractory cGVHD, supporting its use as a viable second-line and steroid-sparing treatment approach.

#### Mycophenolate mofetil

Mycophenolate mofetil (MMF) is rapidly hydrolyzed in vivo to its active metabolite, mycophenolic acid (MPA). The depletion of the guanine nucleotide pool is achieved by selectively inhibiting inosine monophosphate dehydrogenase-II (IMPDH-II), thereby arresting donor effector T cells and self-reactive B cells in the G₁ phase and consequently blocking their clonal expansion [61, 62]. In addition to its role in the downregulation of impaired purine synthesis, MPA has been observed to regulate proinflammatory factors, including IL-2, IL-6, IL-17, and TNF-α. Furthermore, MPA has been demonstrated to weaken T cell co-stimulatory signals, inhibit Th17 polarization, and reduce neutrophil and monocyte recruitment [63]. Furthermore, MPA has been shown to inhibit COX-2 and 5-lipoxygenase, thereby reducing prostaglandin and leukotriene production, and thus rapidly alleviating local inflammation. Concurrently, it has been demonstrated to decrease the release of TGF-β and PDGF, thereby impeding the transformation of fibroblasts into α-SMA+ myofibroblasts [64]. This, in turn, results in a delay in collagen deposition and fibrosis progression in target organs, such as the skin and lungs.

However, the clinical efficacy of MMF in cGVHD varies. A prospective randomized controlled trial conducted by Martin et al. demonstrated that adding MMF to initial systemic therapy for chronic GVHD did not improve outcomes. The success rate for the primary endpoint—resolution of chronic GVHD and discontinuation of all systemic treatment within two years without requiring secondary therapy—showed no significant difference between the MMF and control groups (23% vs. 18%). The trial was terminated early due to a pre-planned interim analysis indicating a very low probability of achieving a positive result for the primary endpoint, and the MMF arm exhibited a trend toward increased mortality risk (HR = 1.99). Accordingly, this study strongly advises against the routine addition of MMF to first-line systemic treatment for chronic GVHD [44].

In contrast, MMF has demonstrated potential in the second-line or salvage setting. In a single-arm study [65], 27 patients with extensive-stage cGVHD who were steroid-resistant/dependent received MMF in combination with sirolimus. The 6-month ORR was 55.6%, and at the 1-year follow-up, the ORR was 59.3%, with progression-free survival (PFS) reaching 62.9% and overall survival (OS) at 100%, suggesting that MMF-based combination therapy may confer clinical benefits in select patient populations.

Of note, MMF-based immunosuppressive regimens are associated with an increased risk of infectious complications. Multiple studies have consistently reported higher incidences of viral infections (particularly cytomegalovirus reactivation) and opportunistic infections in patients receiving MMF-containing regimens [66, 67]. Moreover, intensified immunosuppression may attenuate the graft-versus-leukemia (GVL) effect. Some studies have suggested a potential association between MMF use—especially in combination with other potent immunosuppressive agents—and an elevated risk of disease relapse [68].

Taken together, the available evidence warrants a cautious approach to the use of MMF in cGVHD. Although it may serve as a second-line treatment option in patients with steroid-refractory disease, the lack of benefit in the first-line setting, coupled with the risks of infection and potential relapse, necessitates strict adherence to appropriate indications and close monitoring for adverse events in clinical practice.

#### Sirolimus

Sirolimus exerts its anti-cGVHD effects by inhibiting the mTOR pathway, thereby reducing T-cell and B-cell activation. Furthermore, sirolimus exerts minimal influence on IL-2-dependent Treg expansion and may even upregulate Foxp3 expression, thereby increasing the Treg proportion and facilitating the restoration of immune tolerance [69]. Furthermore, the inhibition of mTORC1-dependent fibroblast proliferation and collagen synthesis results in a reduction of TGF-β and PDGF levels in target organs, consequently decelerating or reversing the fibrotic process. A study of steroid-dependent/resistant cGVHD patients treated with sirolimus combined with a calcineurin inhibitor revealed an ORR of 55.6% after six months [70]. Furthermore, a multicenter randomized phase II/III study further compared the efficacy and safety of different combination regimens. The results demonstrated that the two-drug regimen comprising sirolimus plus prednisone and the three-drug regimen comprising sirolimus, prednisone, and a calcineurin inhibitor showed no significant differences in 6-month complete or PR rates (48.6% vs. 50.0%, *P* = 0.87) or 2-year CR rates (14.7% vs. 15.5%, *P* = 0.90) for the treatment of cGVHD. However, the two-drug regimen exhibited significant advantages in renal function preservation, with lower rates of serum creatinine elevation > 1.5 times baseline at 2 months (1.5% vs. 11.7%, *P* = 0.025) and 6 months (7.8% vs. 24.0%, *P* = 0.016). Based on these long-term follow-up results, prednisone combined with sirolimus is considered an effective alternative for cGVHD treatment, offering easier administration and improved tolerability [45].

Of note, sirolimus is associated with several clinically significant adverse events in real-world applications. The most common toxicities include metabolic abnormalities such as hyperlipidemia, and myelosuppression manifesting as thrombocytopenia and neutropenia [71, 72]. Concurrently, the combination of sirolimus with calcineurin inhibitors substantially increases the risk of thrombotic microangiopathy (TMA) [73, 74]. Other notable adverse events include renal dysfunction, infectious complications, and rare but concerning pancreatitis risk induced by severe hypertriglyceridemia [75]. Importantly, the concurrent use of sirolimus and calcineurin inhibitors exhibits cumulative toxicity, particularly regarding renal impairment and TMA risk. Therefore, when combination therapy is employed in clinical practice, careful risk-benefit assessment and enhanced monitoring are warranted.

#### Azathioprine

Azathioprine exerts its anti-cGVHD effect by inhibiting the proliferation and function of activated T/B lymphocytes through interference with the de novo synthesis pathway of purines. In the initial treatment of cGVHD, azathioprine is commonly combined with prednisone, a regimen which has been demonstrated to reduce steroid failure rates and recurrence [76]. However, it is important to note that this combination carries a high risk of interstitial pneumonia and severe myelosuppression. Of particular concern, the use of azathioprine is associated with a significantly increased risk of secondary malignancies (SM) following HSCT. A nationwide population-based study demonstrated that azathioprine was the single greatest risk factor for SM development after allo-HSCT, increasing cancer risk by 2.55-fold in multivariate analysis (HR 2.55, 95% CI 1.13–5.77; *P* = 0.025). This risk exhibits a clear dose-dependent relationship: compared with lower doses, a cumulative dose exceeding 15,100 mg significantly increased SM risk (HR 3.58 vs. 2.51, *P* = 0.01). The predominant malignancies associated with azathioprine include squamous cell carcinoma (particularly of the oral cavity and skin), esophageal cancer, and lymphoproliferative disorders [77]. The carcinogenic mechanism involves the incorporation of 6-thioguanine nucleotide metabolites into DNA, rendering DNA susceptible to mutagenic oxidation and UVA-induced damage [77–79]. Furthermore, given the paucity of large-scale evidence for refractory cGVHD, azathioprine is not included in standard-of-care regimens for later-line treatment.

#### Thalidomide

The anti-cGVHD effects of Thalidomide are attributable to its capacity for immunomodulation, principally through the rectification of imbalanced immune responses, with additional anti-angiogenic properties that contribute to the alleviation of cGVHD symptoms. Phase II clinical trials indicate that thalidomide monotherapy at 100–160 mg/day is effective in approximately 50% of refractory cGVHD patients [46]. The addition of prednisone or CsA did not enhance efficacy; rather, it resulted in a significant increase in adverse effects, including constipation, insomnia, peripheral neuropathy and haematologic suppression. This led to premature discontinuation in 30%–90% of participants [80]. Consequently, the medication is intended for use as a personalised rescue option, with dosage adjustments made according to efficacy and tolerability.

### Small-Molecule Targeted Therapies

#### Bruton Tyrosine Kinase (BTK) Inhibitors

Ibrutinib represents the first approved oral, irreversible Bruton’s tyrosine kinase (BTK)/IL-2-induced T-cell kinase (ITK) dual inhibitor for the treatment of cGVHD. The substance under scrutiny has been demonstrated to impede the phosphorylation cascade downstream of the B-cell receptor (BCR) and T-cell co-stimulatory signaling. Consequently, this results in the inhibition of B-cell proliferation and auto-antibody production. Furthermore, the substance has been shown to down-regulate Th17 polarization, thereby reducing pro-inflammatory factors such as IL-6, IL-17, and TNF-α. Concurrently, the observed phenomenon is one of a reduction in the levels of chemokines and fibrotic mediators, such as transforming TGF-β and PDGF. This, in turn, results in a reversal of skin and lung collagen deposition [81, 82]. A pivotal Phase Ib/II trial (NCT02195869) demonstrated that, among 42 patients with steroid-refractory/dependent diffuse cGVHD treated with oral ibrutinib 420 mg/day, 67% achieved remission, including 31% with CR. The remission phase persisted for a minimum of 20 weeks in 71% of patients, with 80% demonstrating multi-organ improvement and a total of five patients discontinuing treatment. Adverse events (AEs) were found to be manageable [83]. Subsequent to the findings outlined above, the FDA approved ibrutinib in February 2018 for second-line treatment of adult cGVHD after ≥ 1 prior therapy failure, thereby establishing it as the world’s first targeted therapy for cGVHD. In real-world settings, a multicentre retrospective cohort study (*n* = 270) further demonstrated an 59% failure-free survival rate at 6 months and 41% at 12 months for ibrutinib in treating SR-cGVHD, with significant benefits observed in patients with skin, liver, and oral involvement [84]. Beyond ibrutinib, other BTK inhibitors such as acatinib (NCT04198922) are also actively advancing for cGVHD applications.

#### JAK Inhibitors

In 2021, the FDA approved the use of Ruxolitinib for the treatment of cGVHD in patients aged 12 years and over who had failed at least one prior systemic therapy [85]. Ruxolitinib reversibly binds to the JAK1/2 kinase domain, thereby blocking downstream STAT1/STAT3 phosphorylation of proinflammatory cytokines such as IL-6 and IFN-γ. This results in the inhibition of the proliferation and effector differentiation of donor CD4+/CD8 + T cells, the suppression of Th1/Th17 polarization, and a reduction in the secretion of proinflammatory factors such as IL-17 and TNF-α. The study found that the drug promoted regulatory T cell (Treg) expansion and functional stability, reduced CXCR3-mediated T cell migration to target organs, and decreased fibrotic inflammation in skin, oral mucosa, lungs, and other tissues. In addition, it has been demonstrated that the process under investigation leads to a reduction in collagen deposition and the reversal of tissue fibrosis. This is achieved by means of the downregulation of STAT3-dependent fibroblast activation and TGF-β/PDGF release [86, 87]. The 3-year REACH3 follow-up demonstrated that ruxolitinib provides significant and durable benefits in steroid-refractory cGVHD. Compared with best available therapy (BAT), ruxolitinib extended median failure-free survival from 5.7 to 38.4 months (HR 0.36). The 36-month failure-free survival rates were 56.5% in the ruxolitinib group versus 18.2% in the BAT group, with durable response rates of 59.6% and 26.7%, respectively. These findings confirm that ruxolitinib achieves rapid and sustained disease control with manageable safety, establishing it as the standard second-line targeted therapy for this population [88].

In addition, baricitinib, a selective JAK1/2 (preserving JAK3) inhibitor, has been demonstrated to preserve regulatory T cell function, suppress Th1/Th17 polarization, and accelerate epithelial repair [86]. In a systemic therapy study for ocular cGVHD, baricitinib demonstrated significant improvement in the NIH ocular GVHD score (*P* = 0.014) and exhibited improvements across all ocular surface disease (OSD) parameters, thereby highlighting its potential application in cGVHD [89]. A number of related clinical studies are currently underway (NCT04131738, NCT06475820, NCT02759731). Additionally, JAK-targeted inhibitors such as itacitinib (JAK1-selective) and pacritinib (JAK2/FLT3-dual targeted) are advancing in related studies, with the aim of expanding their use in the treatment of cGVHD.

#### ROCK2 Inhibitors

In 2021, the FDA approved Belumosudil for the treatment of steroid-refractory/dependent chronic GVHD in patients aged ≥ 12 years who have failed ≥ 2 lines of systemic therapy [55, 90]. Belumosudil, a pioneering pharmaceutical agent that functions as a ROCK2 inhibitor, demonstrates its capacity to mitigate the effects of cGVHD by means of two distinct mechanisms: immunomodulation and anti-fibrosis [91]. The pharmaceutical agent belumosudil selectively blocks ROCK2-STAT3 signalling, leading to the downregulation of proinflammatory factors such as IL-17 and IL-21. Concurrently, belumosudil enhances STAT5 phosphorylation, thereby promoting Foxp3 + regulatory T cell expansion and restoring Th17/Treg balance. Inhibition of the RhoA-ROCK2-MRTF axis has been demonstrated to prevent fibroblast-to-α-SMA+ myofibroblast transdifferentiation and reduce TGF-β and PDGF release. This, in turn, has been shown to reverse collagen deposition and fibrosis in target organs such as the skin and lungs. Consequently, Belumosudil provides dual anti-inflammatory and anti-fibrotic benefits, thereby consolidating its fundamental role in precision targeted therapy for cGVHD.

The ROCKstar (NCT03640481) study enrolled 132 patients with cGVHD who had failed at least two prior lines of therapy [92]. The administration of belumosudil resulted in an ORR of 75% (74% with 200 mg once daily, 77% with 200 mg twice daily) and a median duration of response (DOR) of 13.5 months. Observations were made of responses across all affected organs in all subgroups, thus demonstrating high response rates, particularly in cases of lung tissue involvement. The efficacy of the device was further validated by real-world data. A multicentre retrospective cohort study conducted in France (*n* = 68) demonstrated a peak ORR of 57% (CR 15%), with 3- and 6-month ORRs maintained at 47% and 46%, respectively. As demonstrated in Figure, the prevalence of liver and oral remissions was found to exceed 70%, while the incidence of pulmonary remission was approximately 17% [93]. A multicentre study of German and Swiss origin (*n* = 33, median 4 lines) also demonstrated an ORR of 42% in patients with cGVHD receiving a median of 4 lines of prior therapy, with one-third of patients achieving a ≥ 50% reduction in steroid dose [94]. The findings, when considered collectively, demonstrate the efficacy and safety of Belumosudil in refractory cGVHD.

#### Tyrosine Kinase Inhibitors

Imatinib has been shown to treat cGVHD through a combination of antifibrotic and immunomodulatory actions. The substance under scrutiny has been demonstrated to directly reverse organ fibrosis by inhibiting the PDGFR-α/β and TGF-β pathways. In addition, it has been shown to selectively suppress abnormal immune responses by regulating TCR signalling [95, 96]. A multicentre Phase II trial was conducted, enrolling 36 patients with steroid-refractory/dependent cGVHD. The treatment was initiated with oral imatinib at a dosage of 100 mg/day, with the dosage being increased to 400 mg/day, for a period of up to six months. The results demonstrated an ORR of 58.3% (CR: 3 cases, PR: 18 cases), with organ-specific response rates of 70.5% for gastrointestinal involvement, 66.7% for liver involvement, 34.8% for skin involvement, and 25% for lung involvement. Treatment resulted in a significant improvement in patients’ psychological well-being, with 50% of steroid-dependent patients successfully tapering or discontinuing corticosteroids [97]. This finding offers an effective targeted therapy option for patients with multiorgan involvement.

As a second-generation TKI agent, the application of nilotinib in the treatment of cGVHD is also a subject under investigation. Research findings indicate that cGVHD fibroblasts (GVHD-Fbs) demonstrate slow growth characteristics and elevated expression of both COL1α1 (collagen type I alpha 1 chain) and COL1α2. Furthermore, 1 µM Nilotinib has been shown to reduce collagen gene expression in GVHD-Fbs by more than 80%, whilst concomitantly inhibiting intracellular TGF-β and p-Smad2. Following a period of 180 days of Nilotinib treatment, skin biopsies from patients demonstrated a significant reduction in COL1α1/2 staining, accompanied by a return to normal plasma TGF-β levels. It has been demonstrated that Nilotinib exerts its anti-cGVHD effects by means of blocking TGF-β signalling and inhibiting excessive collagen production [98].

However, the clinical application of nilotinib in cGVHD treatment is significantly limited by its adverse effect profile. In a phase I/II dose-escalation and expansion study (NCT01155817), 33 patients with steroid-refractory or steroid-dependent cGVHD received nilotinib, with a maximum tolerated dose of 200 mg/day. At 6 months, the incidence of treatment discontinuation due to drug intolerance was as high as 23%, comparable to the rate of treatment failure attributed to disease progression (23%) [99]. Commonly reported adverse events included hematological toxicities, metabolic abnormalities, cardiovascular events, hepatotoxicity, and infection-related complications [99, 100]. Of note, in a salvage therapy study (NCT02891395), 29 patients who failed prior imatinib treatment were switched to nilotinib. Although some patients achieved responses, up to 79% discontinued treatment due to insufficient efficacy or intolerability, with drug-related adverse events being a primary reason for discontinuation [96]. Furthermore, drug-drug interactions between nilotinib and CYP3A4 inhibitors may lead to elevated nilotinib plasma concentrations, thereby increasing the risk of toxicity. Particular caution is warranted in patients receiving concomitant azole antifungals such as voriconazole or fluconazole following transplantation.

Furthermore, as a second-generation TKI targeted inhibitor, bosutinib has been demonstrated to suppress the expression of GZMK+ CD8 + T cells. In animal models, it has been demonstrated to reduce pulmonary stiffness and fibrosis in cGVHD-BOS mice [101]. While preclinical studies have demonstrated the potential value of bosutinib in cGVHD-BOS, numerous issues—including its efficacy and safety—require further investigation.

#### BCL-2 Inhibitors

BCL-2 inhibitors target anti-apoptotic proteins, and emerging evidence suggests their potential applications in both the prevention and treatment of cGVHD. In the prophylactic setting, preclinical studies have demonstrated that venetoclax, a selective BCL-2 inhibitor, promotes donor chimerism by depleting host natural killer (NK) cells. Jiao et al. established in murine models that recipient NK cells constitute a critical barrier to allogeneic engraftment. By inhibiting BCL-2—an anti-apoptotic protein essential for NK cell survival—venetoclax facilitated efficient donor chimerism under reduced-intensity conditioning (RIC) regimens. Importantly, this approach circumvented the organ toxicity and pro-inflammatory cytokine release associated with myeloablative conditioning (MAC), thereby mitigating the risk of subsequent GVHD development [102].

Regarding therapeutic applications, BCL-2 expression is significantly upregulated in both skin lesions and peripheral blood of patients with cGVHD, with its expression levels closely correlating with transplantation-related mortality and steroid-refractory cGVHD [103]. By inducing T-cell apoptosis, venetoclax may represent a novel targeted therapeutic agent for steroid-refractory cGVHD. However, current clinical evidence supporting venetoclax in cGVHD treatment remains limited to case reports and small case series. Al-Sawaf et al. reported five patients with relapsed chronic lymphocytic leukemia (CLL) following allo-HSCT who received venetoclax in combination with anti-CD20 monoclonal antibody therapy, with no observed exacerbation of GVHD [104]. Similarly, Teoh et al. described a case of relapsed CLL after allo-HSCT complicated by extensive cutaneous cGVHD, in which oral venetoclax administration achieved disease remission without worsening GVHD manifestations [105]. Nevertheless, the direct therapeutic value of venetoclax in steroid-refractory cGVHD warrants validation through large-scale clinical trials.

#### BCL-6 Inhibitors

B-cell lymphoma 6 (BCL-6), a critical transcription factor governing the germinal center (GC) reaction, plays a central role in driving antibody-mediated cGVHD. In terms of prevention, preclinical studies have demonstrated that BCL-6 deficiency in donor T cells or B cells prevents GC formation, thereby abrogating the development of cGVHD. Using a BCL-6 knockout model, Paz et al. confirmed that ablation of BCL-6 expression in donor T cells or B cells prior to transplantation effectively prevents the development of bronchiolitis obliterans (BO)-type cGVHD, significantly reducing collagen deposition and immunoglobulin deposition in lung tissue [106]. These findings suggest that BCL-6 inhibition holds prophylactic potential, capable of blocking cGVHD initiation through early interference with GC responses.

Regarding therapeutic intervention, the small-molecule BCL-6 inhibitor 79 − 6 has demonstrated significant therapeutic potential in established cGVHD animal models. In a murine model of BO with multi-organ involvement, treatment with 79 − 6 reversed established cGVHD, improved pulmonary function (characterized by reduced airway resistance and increased lung compliance), and decreased the number of splenic GC B cells as well as collagen deposition in lung tissue. However, the therapeutic efficacy of 79 − 6 appears to be model-dependent: it was effective in a GC-driven BO model but failed to elicit a therapeutic response in a Th1/Th17-mediated sclerodermatous cGVHD model, indicating that BCL-6 inhibition primarily targets antibody-mediated, GC-dependent cGVHD [106]. An additional study further confirmed that BCL-6 inhibitor 79 − 6 not only suppresses the development and function of peripheral follicular helper T cells (Tfh) and peripheral helper T cells (Tph) but also reduces the number of GC plasma cells and the secretion level of IL-21. This leads to a significant amelioration of clinical manifestations, prolongation of survival, and mitigation of histopathological damage and fibrosis in both skin and lung tissues [107].

Of note, the mechanisms underlying the prophylactic versus therapeutic applications of BCL-6 inhibition differ. Prophylactic application relies on genetic knockout prior to transplantation or early intervention to block GC formation, whereas therapeutic application employs the small-molecule inhibitor 79 − 6 to directly target established GC reactions. Furthermore, the efficacy of therapeutic intervention may vary depending on the disease subtype, with GC-dependent cGVHD exhibiting greater sensitivity to BCL-6 inhibition. Currently, both prophylactic and therapeutic applications of BCL-6 inhibitors in cGVHD remain at the preclinical stage, and their translational value warrants further validation.

#### Proteasome Inhibitors

Bortezomib, a first-generation proteasome inhibitor, has been demonstrated to inhibit NF-κB-dependent transcription, reduce proinflammatory cytokine secretion, induce B-cell apoptosis [108], and suppress germinal centre responses to decrease autoantibody production while blocking antibody-mediated cGVHD pathways [109, 110]. In a prospective Phase I/II trial, investigators explored the utility of bortezomib in allogeneic haematopoietic stem cell transplantation (HSCT) from HLA-unmatched unrelated donors (MMUD). The results demonstrated a cumulative incidence of Grade II-IV acute GVHD at 80 days post-transplant of 22% (95% CI 11%−35%) and a 1-year incidence of chronic GVHD of 29% (16%−43%). With respect to long-term prognoses, the 2-year non-relapse mortality(NRM) rate was found to be 11% (4%−22%), the relapse rate was 38% (24%−52%), and the 2-year progression-free survival and OS rates were recorded at 51% and 64%, respectively. It is noteworthy that MMUD recipients in the bortezomib group exhibited NRM, acute and chronic GVHD incidence, and survival outcomes that were comparable to those observed in patients undergoing HLA-matched reduced-intensity conditioning transplantation at our centre during the same period. In addition, this treatment regimen demonstrated the capacity to stimulate immune reconstitution, as evidenced by the accelerated recovery of CD8 + T cells and NK cells. This study suggests that bortezomib use in MMUD transplantation may achieve survival outcomes comparable to HLA-matched transplantation by regulating immune reconstitution without increasing GVHD risk, offering a viable therapeutic strategy for patients lacking fully matched donors [111].

Ixazomib, an oral second-generation proteasome inhibitor, has been shown to prevent GVHD in preclinical models. Therefore, in order to further evaluate the clinical value of oral ixazomib in preventing cGVHD, a study was conducted that enrolled 51 patients post-HSCT (matched related donors [MRD, *n* = 25] or matched unrelated donors [MUD, *n* = 26]), administering ixazomib on a weekly basis between days + 60 and + 90 post-transplant. The results indicated that, despite the favourable safety and ease of implementation of this treatment regimen (short-term oral administration), the primary clinical endpoint was not met. The 1-year cumulative incidence of cGVHD was 36% in the MRD group and 39% in the MUD group. A comparison of the incidence of cGVHD among recipients of matched related or unrelated donor transplants was conducted with real-world standard-of-care data (CIBMTR). The results indicated that there was no significant reduction in the incidence of cGVHD [112]. However, a recent prospective randomised trial demonstrated that ixazomib significantly reduced moderate/severe cGVHD. The present study comprised 73 patients, with the treatment group receiving 4 mg of ixazomib every 28 days, commencing from day + 100 post-transplant. Statistical analysis revealed that the 1-year cumulative incidence of moderate/severe cGVHD in the ixazomib group was 3.23%, which was significantly lower than the 30.2% observed in the control group (hazard ratio [HR] = 0.089, *p* = 0.02). By the second year, the incidence in the treatment group was only 13%, compared to 43% in the control group (hazard ratio [HR] = 0.23, *p* = 0.01). This study provides compelling evidence to suggest that the administration of ixazomib leads to a significant reduction in the long-term risk of moderate to severe cGVHD [113]. Consequently, the efficacy of ixazomib in the treatment of cGVHD necessitates further validation.

Furthermore, investigators expanded the scope of the study to encompass the impact of carfilzomib on GVHD in the context of unrelated donor hematopoietic stem cell transplantation (SCT) (ClinicalTrials.gov NCT01991301). The present study set out to make a comparison between patients receiving a combination of carfilzomib and cyclosporine/MTX as a prophylaxis regimen, and a historical control group receiving cyclosporine/MTX as a standalone prophylaxis regimen. The results demonstrated that there were no significant differences between the groups in terms of hematopoietic engraftment or treatment-related toxicity. However, the combination group demonstrated a clear advantage in GVHD prophylaxis efficacy: At the 6-month point following the transplantation procedure, the incidence of aGVHD classified as Grade II–IV was 11% (95% confidence interval [CI]: 4–32) in the combination group, which is significantly lower than the 39% (95% CI: 30–50) observed in the control group (*P* = 0.01). Despite the absence of statistically significant differences in the 2-year cumulative incidence of chronic GVHD (49% vs. 41%, *P* = 0.98) and 3-year NRM (11% vs. 18%, *P* = 0.45), the combination group exhibited a tendency towards reduced 3-year relapse rates (8% vs. 26%, *P* = 0.06) and enhanced 3-year OS to 81% (vs. 56% in the control group, *P* = 0.05). The study indicates that the prophylactic regimen of carfilzomib combined with cyclosporine/MTX is well tolerated, significantly reduces the incidence of acute GVHD, and may improve survival outcomes after unrelated donor transplantation. The findings presented here provide a foundation for the subsequent validation of the efficacy of this regimen in larger, controlled trials [114].

#### TGF-β Inhibitors

TGF-β plays a pivotal role in the development of fibrosis in cGVHD by activating fibroblasts and promoting collagen (COL1α1, COL1α2) deposition, resulting in tissue hardening. As indicated by the extant literature, TGF-βinhibitors have been demonstrated to directly suppress the process by obstructing signalling pathways that regulate fibroblast activation and extracellular matrix production. This, in turn, has the potential to decelerate or even reverse fibrosis progression [115, 116]. As a TGF-βinhibitor, tranilast has been demonstrated to alleviate inflammation and fibrosis in animal models by inhibiting the TXNIP and NF-κB signalling pathways, whilst also suppressing epithelial-mesenchymal transition (EMT) processes [117]. Furthermore, clinical observations by Ogawa in 10 patients with cGVHD-associated dry eye demonstrated that topical application of 0.5% Tranilast suspension significantly reduced collagen signaling in the lacrimal glands under confocal microscopy, providing direct clinical imaging evidence for its anti-fibrotic effects [118].

#### Spleen Tyrosine Kinase (SYK) Inhibitors

Spleen tyrosine kinase (SYK) inhibitors can induce B-cell apoptosis in cGVHD patients by blocking the B-cell receptor-BLNK-SYK signaling axis, thereby alleviating manifestations such as skin sclerosis and obliterative bronchiolitis [119]. The pharmaceuticals Fostamatinib and Entospletinib have exhibited efficacy in mouse models and preliminary clinical studies. The collective analysis of the available data provides a robust validation of the therapeutic potential of Fostamatinib, Entospletinib, and analogous agents that target B-cell Syk signalling in the treatment of cGVHD [120]. In a Phase 1 study [121], the clinical value of fostamatinib in preventing and treating cGVHD following allogeneic haematopoietic stem cell transplantation was evaluated for the first time in humans. The results demonstrated not only manageable safety but, more importantly, significant efficacy in treating SR-cGVHD: an ORR of 77%, a CR rate of 31%, and a median duration of response reaching 19.3 months. It is noteworthy that this treatment resulted in a 80% reduction in the median steroid dosage and led to the maintenance of 73% of patients on low-dose therapy after one year, thereby effectively alleviating the burden of long-term steroid use. Furthermore, the study confirmed that fostamatinib selectively eliminates abnormally activated plasma-like cells at an early stage, providing direct evidence for its precise targeting of pathological B-cell pathways in cGVHD. Collectively, these results indicate that fostamatinib offers a novel effective option for the clinical management of cGVHD. Moreover, through its unique SYK inhibition mechanism, it also opens new avenues for understanding and intervening in the pathogenesis of cGVHD. Further confirmatory clinical studies are warranted.

#### Hedgehog Signaling Pathway Inhibitors

Hedgehog inhibitors have been demonstrated to exert a dual anti-fibrotic and immunomodulatory effect through the interruption of the Smo-Gli signalling axis. This results in the direct suppression of fibroblast activation and collagen production, whilst concurrently regulating germinal centre B-cell responses and M2 macrophages [122]. Two Phase I/II clinical trials (GETH-TC and FHD) were conducted to evaluate the efficacy and safety of glasdegib in treating refractory sclerotic cGVHD. The studies enrolled 35 patients with active refractory sclerotic cGVHD who had failed multiple prior therapies. Glasdegib demonstrated significant clinical activity. The GETH-TC trial achieved an ORR of 65% for cutaneous/articular cGVHD at 12 months, while the FHD cohort reached 47%, thus confirming the drug’s efficacy in improving fibrotic lesions in patients unresponsive to conventional treatments. A thorough safety analysis revealed that the primary adverse reactions associated with glasdegib were muscle spasms (Grade 2 in 85% of patients in the GETH-TC trial; Grade 3 in 33% of patients in the FHD trial), alopecia, and dysgeusia. These findings underscore the therapeutic potential of glasdegib in managing refractory scGVHD [123].

#### Transcription Factor RORγt Inhibitors

RORγt is a pivotal transcription factor in Th17 cell differentiation, modulating the expression of inflammatory cytokines such as IL-17. Abnormal Th17 cell activation and elevated IL-17 levels have been observed in cGVHD patients, thus suggesting RORγt as a potential therapeutic target for cGVHD intervention [124, 125]. TMP778 is a first-generation RORγt inhibitor. In a mouse model of cGVHD with obliterative bronchiolitis, TMP778 effectively suppressed the Th17 pathway, significantly reduced inflammatory cytokine production, and successfully alleviated cGVHD disease progression [126]. Despite the encouraging outcomes observed in animal models with RORγt inhibitors, the development of certain candidates for other diseases, such as psoriasis and multiple sclerosis, has been terminated on the grounds of inadequate efficacy or safety concerns [127]. This observation indicates that the utilisation of these agents in cGVHD cases necessitates a meticulous evaluation, with particular emphasis on the potential risks of infection associated with prolonged immunosuppression.

#### Dimethyl Fumarate (DMF)

Dimethyl fumarate (DMF) is a small-molecule compound with immunomodulatory properties, which is currently approved for the treatment of autoimmune diseases such as multiple sclerosis and psoriasis. A preclinical study revealed that DMF effectively suppresses Tfh differentiation and germinal centre responses by activating the transcription factor Nrf2 and downregulating IL-21 transcription. The validity of this effect was confirmed in a mouse cGVHD model and its clinical potential was demonstrated in a human study. The investigation revealed that DMF led to a reduction in the frequency of Tfh cells and IL-21 levels in the peripheral blood of cGVHD patients. This finding indicates that the targeting of Nrf2 to regulate Tfh cells is a pivotal mechanism by which DMF inhibits cGVHD progression, thereby providing both theoretical and experimental evidence for its potential as a novel cGVHD therapy [128] .

#### Glucose Transporter Inhibitors

It has been demonstrated by a number of studies on animals that small-molecule glucose transporter inhibitors (GTIs), such as CG-5 and 2-deoxy-d-glucose (2-DG), have the capacity to inhibit glycolysis in CD4 + T cells. It has been demonstrated that by modulating the metabolic processes of these cells, GTIs (e.g. CG-5 and 2-deoxy-d-glucose [2-DG]) can effectively regulate autoimmune responses in cGVHD models through the inhibition of glycolysis in CD4 + T cells. The metabolic reprogramming process has been demonstrated to have several important functions in the human body, including the restriction of excessive activation and proliferation, the suppression of pathogenic Th1/Th17 cells, and the reduction of autoantibody production [129, 130]. Presently, the implementation of GTIs in cGVHD remains in the preclinical research phase. The objective of this strategy is to regulate immunity at the metabolic level, thus offering a novel perspective for deepening understanding and treating cGVHD. Nevertheless, the clinical benefits of this approach require further validation and exploration through subsequent studies.

### Cytokine Therapy

#### IL-2 Therapy

The fundamental mechanism of low-dose IL-2 therapy for cGVHD is the selective activation and expansion of Tregs, thereby restoring immune balance and suppressing excessive autoimmune responses [131]. In preliminary studies, Koreth [132]et al. initially reported the treatment outcomes for 29 patients suffering from SR-cGVHD who were treated with interleukin-2 (IL-2). The study evaluated three dose levels: 0.3, 1.0, and 3.0 million IU/m². The majority of patients enrolled in the study demonstrated multi-organ involvement, with a median number of three organs affected. Among the 28 patients evaluable for toxicity, no recurrence or progression of cGVHD or underlying malignancy was observed. A thorough safety analysis was conducted, which identified the maximum tolerated dose of IL-2 to be 1 million IU/m² on a daily basis. In the highest dose group (3 million IU/m² daily), systemic symptoms requiring dose reduction led subsequent studies to adopt 1 million IU/m² daily as the safe and effective therapeutic dose. Furthermore, a study involving 33 patients with severe-relapse cGVHD demonstrated that low-dose IL-2 (1.0 million IU/m²) achieved an ORR of 61% (20/33), with organ involvement in the skin, liver, gastrointestinal tract, lungs, and joints all showing improvement. Subsequent mechanistic studies corroborated the hypothesis that low-dose IL-2 selectively expands Tregs, increasing their numbers by more than fivefold whilst simultaneously boosting NK cells by over fourfold. Conventional T cells (Tcons) remained unaltered. This finding serves to substantiate the hypothesis that the mechanism of action in question pertains to precise immune modulation. Furthermore, predictive analyses revealed a strong correlation between treatment timing and clinical response: earlier initiation of IL-2 therapy after transplantation or cGVHD onset correlated with superior efficacy [133]. A pre-treatment Treg/Tcon ratio of at least 0.07, in combination with a ratio of at least 0.2 within the first week of therapy, has been shown to serve as an effective predictor of clinical remission.

Subsequent real-world studies have further validated the efficacy of IL-2 in cGVHD. As reported by Wobma H et al. [134], the administration of low-dose IL-2 (1 × 10⁶ IU/m²/day) resulted in notable therapeutic efficacy in 13 paediatric and young adult patients diagnosed with SR-cGVHD, who had completed a minimum of 4 weeks of treatment. The ORR was 85% (11/13 patients, including 5 CR and 6 PR), with responses involving multiple affected organs. Furthermore, immunological markers demonstrated Treg-dominant expansion, with a median peak fold increase of 2.8 in the Treg-to-CD4 + conventional T cell ratio. The drug’s definitive efficacy and favourable safety profile mean that low-dose IL-2 offers an important new clinical treatment option for SR-cGVHD.

### Monoclonal Antibody Therapy and Biologic Therapy

#### Abatacept

Abatacept is a CTLA-4-Ig fusion protein that competitively binds CD80/CD86 on antigen-presenting cells (APCs) with high affinity, thereby blocking their interaction with T-cell CD28. It has been established that this process prevents T cells from achieving full activation due to the absence of co-stimulatory signals. This, in turn, results in their entry into an incompetent or apoptotic state, thereby suppressing the pathological progression of cGVHD [135]. The efficacy of the T-cell co-stimulation blocker abatacept was initially determined through a Phase I study. The results obtained demonstrated a satisfactory level of safety, with a 44% PR observed in 16 patients and a significant reduction in steroid dosage (the mean prednisone dose decreased by 51.3%) [136]. Subsequent Phase II extension study (NCT01954979) built on these positive signals, demonstrating an increased ORR of 58% for abatacept in treating SR-cGVHD. The treatment demonstrated favourable safety, accompanied by down regulation of key inflammatory mediators and PD-1 expression on CD4 + T cells. This reshaped the immune microenvironment and further validated the efficacy of abatacept. Abatacept is a significant advancement towards precision-targeted immunomodulatory therapy for cGVHD. Its clinical value has been progressively validated and warrants further research and implementation [137].

#### Anti-CSF-1R Monoclonal Antibodies

Axatilimab is a humanised IgG4 monoclonal antibody that exhibits high affinity binding to the colony-stimulating factor-1 receptor (CSF-1R) on the surface of monocytes and macrophages. It achieves this by competitively binding to and thereby blocking the binding of its ligands, CSF-1 and IL-34. This results in the inhibition of receptor dimerisation and downstream JAK2/STAT3 phosphorylation, consequently leading to a reduction in the secretion of pro-inflammatory and pro-fibrotic factors, including TGF-β, PDGF, IL-6, and IL-17. Furthermore, depletion of macrophages has been demonstrated to exert a marked effect on the immune system, specifically by weakening Th17 polarization and B-cell activation, promoting Foxp3 + regulatory T-cell expansion, and restoring Th17/Treg balance. This, in turn, has been shown to exert an anti-cGVHD effects [138–140]. In an international multicentre randomised open-label Phase II study, 241 patients with relapsed/refractory cGVHD who had failed ≥ 2 prior lines of therapy were randomised to receive 0.3 mg/kg Q2W, 1 mg/kg Q2W, or 3 mg/kg Q4W. The results demonstrated that the low-dose axatilimab group (0.3 mg/kg administered every two weeks) achieved an ORR of 74%, with a median response duration of 18 months and favourable safety profile. This established the dose as the recommended regimen for subsequent use [141]. Subsequent to the analysis of these results, the FDA formally approved Axatilimab in August 2024 for patients ≥ 12 years of age, weighing ≥ 40 kg, with cGVHD who have failed at least two prior lines of therapy [142].

#### Anti-CD20 Monoclonal Antibodies

Rituximab is a chimeric anti-CD20 monoclonal antibody that rapidly eliminates B cells via antibody-dependent cellular cytotoxicity (ADCC), complement-dependent cellular toxicity (CDC), and apoptosis induction. This action has been demonstrated to reduce autoantibody production, attenuate Th1/Th17 polarization and associated proinflammatory cytokine release, and inhibit BAFF-mediated fibroblast activation. Consequently, these effects exert a therapeutic and reversal effect on cGVHD, particularly cutaneous and fasciitis [143]. In a Phase II prospective study of patients with newly diagnosed cGVHD who had undergone HSCT (NCT01135641) [144], rituximab (375 mg/m²weekly for 4 weeks) in combination with corticosteroids and cyclosporine A was utilised as the initial treatment regimen. The results demonstrated a high 1-year treatment response rate of 83%, with 74% of evaluable patients successfully weaning off corticosteroids. The 1-year OS rate of 83% substantiates the hypothesis that this combination regimen effectively controls cGVHD and improves clinical outcomes.

Furthermore, in patients with steroid-refractory cGVHD, several retrospective studies and small-sample prospective studies have demonstrated that rituximab, administered at either standard or low-dose regimens, achieves an ORR of approximately 50%–70% and facilitates the reduction of immunosuppressants in a subset of patients [143, 145, 146]. A subsequent systematic review included seven studies—comprising 111 patients, with three non-controlled prospective studies and four retrospective studies—and demonstrated a pooled ORR of 66% (95% CI 57%–74%) for rituximab in cGVHD. Organ-specific analyses revealed response rates of 60% for skin involvement, and 36%, 29%, 31%, and 30% for oral, hepatic, gastrointestinal, and pulmonary involvement, respectively. Response rates for ocular and musculoskeletal involvement ranged from 13% to 38% and 75%–100%, respectively. The pooled mortality rate was 15.8% (95% CI 8.3%–25.3%), and several studies reported a median glucocorticoid dose reduction of 75%–86%. However, this analysis was predominantly based on small-sample, non-randomized studies, with inherent risks of selection bias and analytical bias, resulting in limited overall evidence quality. Regarding safety, common adverse events included infusion reactions and infectious complications [147]. In summary, rituximab demonstrates modest efficacy in steroid-refractory cGVHD; nevertheless, its long-term survival benefits and optimal treatment strategy warrant further validation through high-quality randomized controlled trials.

Ofatumumab is a fully humanised monoclonal antibody that targets CD20. The presence of the CD20 circular epitope is recognised (in contrast to rituximab), and this has been shown to enhance CDC and ADCC activity. The consequence of this is that it facilitates complete B-cell depletion and elimination of germinal centre response [148]. A Phase II study was conducted on 38 patients suffering from cGVHD. The study evaluated the potential of ofatumumab combined with prednisone for treating cGVHD. The study demonstrated that this combination regimen is safe, and that it is more effective than historical controls, as indicated by a post-hoc analysis: a 6-month ORR of 62.5%, and a significantly higher probability of achieving steroid-free status at 24 months among responders. The findings indicate that the early administration of ofatumumab may provide clinical benefits for patients diagnosed with cGVHD [43].

Preliminary studies suggest the potential clinical value of CD20 monoclonal antibodies in the treatment of cGVHD, but further validation is required through large-scale randomised controlled trials to ascertain their therapeutic advantages. Furthermore, pivotal parameters such as optimal dosage, treatment cycles, and the timing of administration require further definition. Additionally, the risk of HBV reactivation and other infectious events following administration necessitates ongoing monitoring and assessment.

#### Anti-CD25 Monoclonal Antibody

Anti-CD25 monoclonal antibodies represent a widely utilised second-line therapeutic agent within clinical practice. The mechanism of action of these agents principally involves the blocking of the IL-2/IL-2Rα signalling pathway, the selective inhibition of the proliferation of activated T cells, the reduction of Th1/Th17 cell polarisation, and the indirect downregulation of B cell activation and antibody production. This process serves to alleviate inflammatory responses that are mediated by T and B cells [149]. Currently, the most commonly used CD25 monoclonal antibodies are daclizumab and Basiliximab.

In the field of cGVHD prophylaxis, a recent prospective study has provided important evidence supporting the use of anti-CD25 monoclonal antibodies. Zhang et al. investigated a humanized anti-CD25 monoclonal antibody as a substitute for conventional MTX-based prophylaxis in haploidentical HSCT. Compared with a single-dose regimen (25 mg/day anti-CD25 mAb combined with MTX), a double-dose regimen (50 mg/day without MTX) not only significantly reduced the incidence of grade III–IV acute GVHD (7.35% vs. 18.42%, *P* = 0.047), but also markedly decreased the occurrence of chronic GVHD (20.59% vs. 40.01%, *P* = 0.006) and moderate-to-severe cGVHD (10.29% vs. 30.84%, *P* = 0.001) [150]. In addition, the double-dose regimen was associated with a lower incidence of oral mucositis and accelerated hematopoietic recovery, while OS outcomes were comparable between the two groups. Although this study was primarily designed to optimize post-transplant immunosuppressive strategies, its findings suggest that anti-CD25 monoclonal antibodies are not only biologically plausible in immune modulation but may also contribute to reducing the risk of cGVHD.

At the therapeutic level, direct evidence for the use of anti-CD25 monoclonal antibodies in cGVHD remains limited, with most studies consisting of small exploratory cohorts or retrospective analyses. Among these, daclizumab has failed to demonstrate satisfactory efficacy and has been associated with an increased risk of infections [151]. In contrast, basiliximab has shown some therapeutic potential in cGVHD. In a retrospective study including 41 patients with gastrointestinal cGVHD, the CR rate in the basiliximab group (*n* = 25) reached 88.0%. No statistically significant differences were observed between the basiliximab and non-basiliximab groups in terms of OS, relapse rate, or NRM, and no new severe hepatic or renal dysfunction or cytokine release syndrome(CRS) was reported. The incidence of hematologic and infection-related adverse events was comparable to that observed with other systemic immunosuppressive agents [152].

In summary, current evidence suggests that anti-CD25 monoclonal antibodies may have potential value in both the prevention and treatment of cGVHD. However, these conclusions are largely derived from indirect evidence or retrospective analyses, and further large-scale, prospective studies specifically designed for cGVHD are warranted to validate their efficacy and safety.

#### Anti-IL-26 Monoclonal Antibody

IL-26, a Th17 cytokine, has been identified as playing a pivotal role in the chronic inflammation of cGVHD. Research has demonstrated that IL-26 has a role in promoting tissue damage, with studies showing an increase in neutrophil levels and Th17 cytokine expression when IL-26 is present [153]. A humanised anti-IL-26 monoclonal antibody has been demonstrated to possess significant therapeutic efficacy in chronic xenogeneic GVHD models. This efficacy is characterised by a reduction in weight loss and an increase in survival duration, whilst simultaneously preserving GVL effects [154]. This offers a novel direction for targeted therapy in cGVHD.

#### Anti-CD38 Monoclonal Antibody

Daratumumab (Dara), an anti-CD38 monoclonal antibody, is a widely utilised agent in the therapeutic management of multiple myeloma. It has been demonstrated that Dara exerts its effects by inhibiting CD8 + T cell proliferation/activation, downregulating inflammatory factors, and promoting immunosuppressive T cells. Concurrently, metabolic reprogramming is employed to preserve anti-leukemic effects, including Th17 and Tc1/17 responses, thereby achieving the separation of anti-GVHD effects from GVL effects. In humanised mouse models, Dara-treated experimental groups exhibited significant improvement in allogeneic GVHD (reduced weight loss, prolonged survival and lower scores) [155]. A number of observational clinical studies have indicated that, among 34 multiple myeloma patients who received Dara following an allogeneic haematopoietic stem cell transplantation, only five patients (15%) developed aGVHD following Dara treatment. No cGVHD events were subsequently observed [156]. These findings suggest that daratumumab may have the potential to be both safe and efficacious in the treatment of GVHD.

#### Anti-BAFF/APRIL Monoclonal Antibodies

Belimumab is a fully human IgG1λ monoclonal antibody that targets soluble B-lymphocyte stimulating factor (BAFF/BLyS). It has been approved by the FDA for the treatment of active systemic lupus erythematosus [157]. The BAFF blockade has been demonstrated to disrupt the immunopathogenic cycle in two ways: firstly, by eliminating pathogenic B cells and suppressing germinal centre responses and Tfh cells, and secondly, by indirectly modulating T cells (reducing Th17, increasing Treg). Currently, the application of belimumab in the field of cGVHD remains in the early stages of exploration. Jia et al. reported that elevated BAFF levels in cGVHD result from high BAFF expression in recipient fibroblastic reticular cells and donor-derived CD4 + T cells. BAFF promotes the persistence of B cell receptor (BCR)-activated B cells and the production of alloantibodies by upregulating NOTCH2 expression on B cells and enhancing BCR signaling, thereby driving the pathogenesis of cGVHD [158], suggesting that targeting the BAFF and BCR pathways represents a potential therapeutic strategy for cGVHD. Furthermore, studies have shown that early blockade of BAFF after allo-HSCT in mouse models may modulate CD4 + T cell-induced acute GVHD while also exhibiting the potential to control cGVHD that may develop at later stages [159]. Therefore, the potential role of belimumab in cGVHD remains under investigation, and its clinical value awaits validation by future prospective studies. At present, it cannot be recommended as an evidence-based treatment option.

#### Anti-TNF-α Monoclonal Antibodies

Infliximab is a chimeric human-mouse IgG1 anti-TNF-α monoclonal antibody that neutralizes both soluble and membrane-bound forms of TNF-α, thereby inhibiting its mediated pro-inflammatory cascade, including vascular endothelial activation, leukocyte recruitment, and tissue injury [160]. In the context of cGVHD, evidence for anti-TNF-α therapy remains limited, primarily derived from small-sample retrospective studies and case series. For instance, Kamachi et al. reported a case of a 21-year-old female who developed intestinal GVHD with typical Crohn’s disease features approximately one year after umbilical cord blood transplantation; the patient was refractory to steroid therapy but achieved rapid symptom improvement following treatment with infliximab and adalimumab, with no recurrence after discontinuation [161]. In another retrospective study, three patients with steroid-refractory cGVHD all achieved clinical remission after receiving infliximab; however, all experienced infections, highlighting the associated infectious risk [162]. Furthermore, a prospective trial demonstrated that the addition of infliximab to standard GVHD prophylaxis failed to reduce the incidence of GVHD but significantly increased the risk of bacterial and invasive fungal infections [163]. In summary, although TNF-α plays a critical role in the pathogenesis of GVHD, the application of anti-TNF-α monoclonal antibodies in cGVHD currently lacks support from high-quality prospective clinical trials, and their role is more appropriately positioned as a salvage therapy option. Clinical use should be based on careful assessment of individual patient risks (infection, relapse of primary disease) and potential benefits, implemented in experienced centers, and accompanied by enhanced infection surveillance and prophylactic measures.

#### Anti-CD52 Monoclonal Antibody

Alemtuzumab is a humanised anti-CD52 monoclonal antibody that rapidly binds to T/B cells, certain NK cells, and dendritic cells expressing high levels of CD52. The rapid lysis of these cells is achieved via CDC and ADCC [164], thereby exerting the anti-cGVHD effect. The efficacy of alemtuzumab monotherapy in treating cGvHD has been evaluated in a Phase I clinical study. In 13 patients with steroid-refractory cGvHD, the maximum tolerated dose of alemtuzumab was determined as Dose Level 2 (3 mg on day 1, followed by 10 mg on days 3, 5, 8, 15, and 22). Among the 10 evaluable patients, 70% achieved a response at 12 weeks (30% complete remission); four patients reduced immunosuppressants, with a median prednisone reduction of 61.6% at one year. Although alemtuzumab monotherapy demonstrated activity in heavily pretreated cGvHD, infectious complications increased with higher doses, emphasizing the importance of stringent prophylaxis and monitoring [165].

Furthermore, Low-dose alemtuzumab combined with rituximab has demonstrated synergistic efficacy in steroid-refractory cGvHD. In a prospective study (NCT01042509) of 15 patients, the regimen (alemtuzumab 10 mg/day, days 1–3; rituximab 100 mg/day, days 4, 11, 18, 25) achieved a 100% ORR at 30 days (33% complete, 67% partial), with sustained responses at 90 days. This combination enables rapid disease control via concurrent T- and B-cell targeting [166].

In summary, the application of alemtuzumab in cGVHD treatment includes both monotherapy and combination regimens. Monotherapy requires dose optimization to balance efficacy against infection risk. Combination with rituximab may enhance efficacy, though infection risk remains a concern. Current evidence is derived primarily from small-sample prospective studies, and larger randomized controlled trials are warranted to establish its optimal therapeutic role.

#### Anti-IL-6 Monoclonal Antibodies

The suppression of the STAT3-RORγt axis, the downregulation of Th17 cells, the upregulation of Tregs, and the reduction of autoantibodies and fibrosis are all effects of IL-6 monoclonal antibodies [167]. A number of studies have demonstrated the significant therapeutic potential of tocilizumab. For example, in an SR-GVHD cohort (acute, *n* = 6; chronic, *n* = 2), approximately 67% of patients with acute GVHD achieved an ORR within 56 days, while patients with cGVHD also exhibited marked responses or stable disease. Furthermore, immunosuppressants were successfully tapered and discontinued [168]. The results of the study indicate that this drug is a promising therapeutic option, warranting further investigation. Furthermore, a prospective paediatric cohort (*n* = 6) demonstrated a 100% response rate with tocilizumab plus ruxolitinib, with a median time to response of 26.7 days. Patients with recurrent chronic cutaneous cGVHD achieved sustained remission without new infections [169]. Whilst the findings of these small-sample studies suggest the existence of therapeutic potential for anti-IL-6 monoclonal antibodies, further validation is required through large-scale randomised controlled trials in order to ascertain the precise efficacy of the aforementioned antibodies and their long-term safety.

#### Others (Preclinical Research Stage)

Anti-ICOS Monoclonal Antibody: ICOS (Inducible T-cell COStimulator) is a key co-stimulatory molecule for T-cell activation. The administration of anti-ICOS monoclonal antibodies has been demonstrated to impede this pathway, thereby enhancing cytokine release and infiltration. In a canine cGVHD model, the administration of an anti-ICOS monoclonal antibody resulted in clinical remission, as indicated by a marked reduction in skin or gingival lesions, in four out of five treated dogs. Furthermore, the median survival period was extended from 10 days in the historical control group to 30 days [170].

Anti CX3CL1 Monoclonal Antibody: CX3CL1 is a key chemokine that guides immune cells expressing its receptor CX3CR1 (e.g. macrophages, T cells) to migrate towards tissues, playing a crucial role in inflammation and fibrosis. The administration of an anti-CX3CL1 monoclonal antibody during the fibrotic phase of Scl-cGVHD resulted in a significant suppression of skin and lung fibrosis, without the occurrence of notable adverse reactions [171]. This provides robust preclinical evidence for targeting the CX3CL1-CX3CR1 signaling pathway to treat chronic skin and lung fibrosis in GVHD, supporting its advancement to clinical trials.

### Cellular Immunotherapy

As our understanding of the pathogenesis of cGVHD has deepened, the functional imbalance of key immune cells, such as Th17 and Treg, has emerged as a core driver of cGVHD progression. Consequently, the development of novel cell immunotherapies has emerged as a breakthrough in this field, promising to unveil new avenues for the precision treatment of cGVHD.

#### Mesenchymal Stem Cells

Mesenchymal stem/stromal cells (MSCs) are a type of multipotent adult stem cell derived from the mesoderm, possessing self-renewal and multipotent differentiation potential. Initially identified in bone marrow, these cells have subsequently been isolated from various tissues, including umbilical cord, adipose tissue, dental pulp, placenta, and amniotic fluid. Research indicates that IFN-γ-activated MSCs have the capacity to deplete tryptophan by expressing indoleamine 2,3-dioxygenase (IDO), directly inducing T-cell cycle arrest and suppressing proinflammatory cytokine release. Concurrently, they regulate the BAFF/BAFF-R axis to reduce autoantibody production. It has been demonstrated that these dual pathways can synergistically mitigate tissue inflammation and immune complex-mediated fibrosis [172].

In the field of cGVHD prophylaxis, a multicenter randomized controlled trial (*n* = 148) conducted by Chinese investigators demonstrated that early repeated infusions of umbilical cord-derived MSCs (1 × 10⁶ cells/kg, once every two weeks for a total of four infusions) following allo-HSCT significantly reduced the incidence of severe cGVHD (5.4% vs. 17.4%, *P* = 0.03) and improved GVHD-free, relapse-free survival [173].

At the therapeutic level, a prospective Phase I clinical trial was conducted to evaluate the efficacy of MSCs in SR-cGVHD. Ten patients with SR-cGVHD received a total of four intravenous infusions (1 × 10⁶ cells/kg each) every two weeks. The ORR at eight weeks after the first infusion was 60%, with general improvements observed in clinical symptoms and quality of life [174].

Currently, the application of MSCs in cGVHD remains in the clinical research stage and is primarily recommended for use in clinical trials or compassionate use settings. The precise efficacy, optimal dosage, and infusion regimen require further validation through large-scale randomized controlled trials.

#### Treg Cell Therapy

A significant reduction in peripheral blood and target organ Tregs has been observed in cGVHD patients. In the course of in vitro expansion or reinfusion of donor-derived Tregs, there is a rapid increase in the Treg/effector T cell (Teff) ratio, thus restoring immune balance and exerting anti-cGVHD effects [175]. Trzonkowski P et al. were the first to undertake a clinical trial in which they explored the potential of ex vivo expanded human CD4 + CD25+CD127- regulatory T cells as a treatment for GVHD. Following infusion, one of two cGVHD patients demonstrated resolution of symptoms and successful tapering/discontinuation of immunosuppression. No instances of DLT, infection, or recurrence were observed. This initial trial demonstrated the potential value of Treg cell therapy in controlling cGVHD [176]. In a Phase I study [177], researchers firstly evaluated the safety and immunological effects of the combined infusion of donor-derived Treg-DLI with low-dose IL-2 in 25 adult patients suffering from SR-cGVHD. The results demonstrated that 20% of patients experienced a PR after eight weeks of treatment, while an additional 40% of patients achieved disease stabilisation, without affecting CD4+/CD8 + conventional T cell subsets. TCR sequencing further revealed enhanced Treg diversity post-treatment, with some donor-derived Treg clones persisting for over one year. The present study establishes a safety and mechanistic foundation for the clinical application of adoptive Treg therapy in SR-cGVHD. It is important to note that related clinical trials (e.g., NCT03198234) are currently underway to provide further validation of the efficacy and safety of the aforementioned treatment.

#### CAR-T/NK Cell Therapy

Presently, the exploration of Chimeric Antigen Receptor(CAR) T/NK cell therapies for cGVHD remains in the proof-of-concept phase. In a significant development, Hisashi Yano and his associates have successfully devised a method to directly induce CD4 + regulatory T-like cells (iCD4 + Treg-like cells) from human induced pluripotent stem cells (hiPSCs). By inducing high-level FOXP3 expression in this differentiation system, the resulting cells lost their ability to secrete inflammatory cytokines and exhibited Treg-specific epigenetic features along with stable immunosuppressive functions. Subsequent studies revealed that HLA-A2 CAR-modified iCD4 + Treg-like cells effectively suppressed allogeneic CD8 + T cell proliferation in vitro and exhibited disease control comparable to natural Tregs in a mouse xenogeneic GVHD model. This research provides a novel technical pathway for developing precise, renewable cell therapies for cGVHD [178]. In addition, the research team led by Yongxian Hu team investigated a novel sequential treatment regimen in 10 patients with relapsed/refractory CD7-positive haematological malignancies. The treatment regimen involved the induction of remission with CD7 CAR-T cell therapy, followed by allo-HSCT without myeloablative conditioning or GVHD prophylaxis. The results demonstrated only three cases of Grade 2 acute GVHD, with no severe GVHD observed. With a median follow-up period of 15.1 months, the majority of patients demonstrated sustained remission, exhibiting a 1-year OS rate of 68%. This study provides indirect evidence that CD7-targeted CAR-T cell therapy may be a viable approach for managing GVHD [179].

### Extracorporeal photopheresis

Extracorporeal photopheresis (ECP) is a therapeutic modality that modulates immune responses by isolating peripheral blood mononuclear cells, sensitizing them with 8-methoxypsoralen (8-MOP) and exposing them to ultraviolet A irradiation prior to reinfusion. Its primary mechanisms include the induction of aberrant T cell apoptosis, expansion of regulatory T cells (Tregs), and modulation of dendritic cell function, thereby promoting immune tolerance [180, 181]. ECP has been widely applied in the management of cGVHD, particularly in patients with glucocorticoid-resistant or -dependent disease.

Real-world studies have supported the clinical value of ECP as monotherapy. For instance, Greco et al. evaluated 75 patients with steroid-refractory or multiply pretreated cGVHD and demonstrated that the ORR increased progressively over time (21% at 3 months, 57% at 6 months, and 70% at 12 months), with a 12-month overall survival rate of 85.9% and a significant corticosteroid-sparing effect, suggesting sustained and cumulative clinical benefits [182]. Furthermore, combination strategies for refractory cases have attracted increasing attention. Studies have shown that ECP combined with the JAK1/2 inhibitor ruxolitinib yielded an ORR of approximately 74% (CR 9%, PR 65%) in cGVHD, indicating potential synergistic immunomodulatory effects [183].

In addition, multiple systematic reviews and meta-analyses showed that the pooled 12-month OS and failure-free survival rates in patients treated with ECP were 83.97% and 60.79%, respectively. The ORR was 45.34% at 3–4 months of treatment and increased to 58.23% at 6–8 months. The skin-specific response rate was 34.86% at 2–3 months and reached 54.22% at 4–6 month [184]. Collectively, ECP constitutes a significant and promising immunomodulatory approach in the management of steroid-refractory cGVHD. The treatment agents and recommendations for cGVHD are summarized in Table 1.

**Table 1:** Therapeutic agents for chronic GVHD

category	Agent	Mechanism	Evidence level	Key evidence
First-line Standard Therapy				
Glucocorticoids	Prednisone	Broad immunosuppression	Standard of care	6-month ORR 48.6%−62.5%,
Calcineurin inhibitors	Cyclosporine A, Tacrolimus	Inhibition of T-cell activation	Standard of care	Combined with steroids as first-line regimen
Approved targeted therapies				
	Ruxolitinib	JAK1/2 inhibitor	Phase III RCT/FDA-approved	REACH3 trial superior FFS vs. BAT
	Ibrutinib	BTK/ITK inhibitor	Phase Ib/II/FDA-approved	NCT02195869: ORR 67%
	Belumosudil	ROCK2 inhibitor	Phase II/FDA-approved	ROCKstar trial: ORR 75%
	Axatilimab	Anti-CSF1R mAb	Phase II RCT/FDA-approved (2024)	ORR 74%
Conventional immunosuppressants				
	MTX	Antimetabolite	Phase II/retrospective	ORR ~ 70% (small studies)
	MMF	IMPDH inhibitor	RCT (negative) + retrospective	No benefit in frontline (Martin et al.)
	Sirolimus	mTOR inhibitor	Phase II/III randomized	No superiority vs. standard
Monoclonal antibodies				
	Rituximab	Anti-CD20	Phase II + meta-analysis	ORR ~ 66%
	Ofatumumab	Anti-CD20 (different epitope)	Phase II clinical trial	6-month ORR 62.5%
	Abatacept	CTLA4-Ig	Phase I/II	ORR ~ 58%
	Basiliximab	Anti-CD25	Retrospective	Limited evidence
	Alemtuzumab	Anti-CD52	Phase I dose-escalation	70% ORR (30% CR)
	Infliximab	Anti-TNF-α	Retrospective case series	Case reports; RCT for prophylaxis failed
	Tocilizumab	Anti-IL−6R	Small sample prospective	ORR ~ 67%; 100% response when combined with ruxolitinib
	Daratumumab	Anti-CD38	Observational study (MM patients)	34 post-allo-HSCT patients; cGVHD incidence 15%
	Belimumab	Anti-BAFF	Preclinical/early clinical	No prospective cGVHD trials; mechanistic studies show potential
Small Molecule Inhibitors				
	Imatinib	PDGFR/TGF-β inhibition	Phase II	ORR ~ 58%
	Nilotinib	TKI	Phase I/II	Limited by toxicity
	Carfilzomib	Proteasome inhibition	Phase I/II	Limited clinical data
	Bortezomib	Proteasome inhibition	Phase I/II	Limited clinical data
	Fostamatinib	SYK inhibitor	First-in-human/Phase I	ORR 77%
	Baricitinib	JAK1/2 inhibitor	Early clinical studies	Small cohorts
	BCL-2 inhibitors (Venetoclax)	Apoptosis induction	Case reports/preclinical	Limited clinical data
	BCL-6 inhibitors	GC reaction inhibition	Preclinical	Animal models only
	RORγt inhibitors	Th17 pathway	Preclinical	Animal models
Cellular/immunomodulatory therapy				
	ECP	Immune tolerance induction	Prospective + real-world studies	ORR ~ 60–70% (meta-analysis + cohort)
	Low-dose IL-2	Treg expansion	Phase I/II	ORR ~ 60%
	MSCs	Immune modulation	Early-phase/heterogeneous	Small studies, inconsistent results
	CAR-T/NK cells	Engineered cells	Targeting CD7, etc.	Preclinical

*ORR* overall response rate, *MTX* methotrexate, *MMF* mycophenolate mofetil, *ECP* extracorporeal photopheresis, *MSCs* mesenchymal stem/stromal cells, *CAR* chimeric antigen receptor

## Discussion

First-line therapy for cGVHD remains predominantly centered on systemic corticosteroids, with or without CNIs. Corticosteroids exert broad-spectrum immunosuppressive effects and enable rapid control of inflammatory manifestations, establishing them as the standard initial treatment. However, long-term corticosteroid use is associated with significant toxicities. Moreover, a subset of patients develops steroid-refractory or steroid-dependent disease, highlighting a major limitation of first-line therapy. The addition of calcineurin inhibitors may enhance immunosuppressive efficacy and improve response rates in certain patients, but it also increases the risks of nephrotoxicity, hypertension, and opportunistic infections, without fully overcoming steroid resistance.

In recent years, second-line treatment options have expanded substantially, offering more targeted therapeutic strategies. Among these, JAK inhibitors have demonstrated favorable efficacy in steroid-refractory cGVHD by modulating multiple inflammatory cytokine pathways, although their use may be associated with cytopenias and an increased risk of viral reactivation. Similarly, BTK inhibitors target B-cell signaling and have shown efficacy in patients with prominent B-cell involvement; however, ORR are variable, and adverse events such as infection and bleeding remain important considerations. Belumosudil, a ROCK2 inhibitor, maintains immune homeostasis by regulating Th17/Treg differentiation and fibrotic pathways, representing another promising agent. In heavily pretreated patients, this agent has demonstrated encouraging efficacy, particularly in those with pulmonary involvement. Nonetheless, long-term data and direct comparisons with other second-line agents remain lacking. In addition to pharmacological therapies, cellular approaches such as MSCs and ECP exert immunomodulatory effects with relatively lower systemic toxicity and may serve as monotherapy or combination strategies. These modalities may be particularly suitable for patients intolerant to intensive immunosuppression, although challenges persist regarding their accessibility, cost, and heterogeneity in treatment response.

Overall, first-line therapies are characterized by rapid onset but limited durability and substantial toxicity, whereas second-line therapies offer greater specificity and the potential for more sustained responses, albeit often associated with higher costs and slower onset of action. Furthermore, response rates vary considerably across different agents depending on the involved organs. Therefore, in the management of refractory cGVHD, treatment strategies should be individualized based on disease severity, organ involvement, prior treatment response, and patient comorbidities.

### Summary

The treatment paradigm for cGVHD has undergone a fundamental shift, evolving from traditional non-specific immunosuppression towards precision medicine centred on targeted therapies treatments. At present, a number of innovative therapeutic strategies have been shown to demonstrate encouraging efficacy and reliable safety profiles in clinical practice. Within the domain of immunomodulatory therapy, low-dose IL-2 has been demonstrated to effectively promote the restoration of immune tolerance by specifically expanding regulatory T cells. The use of small-molecule inhibitors that target key signalling pathways, including JAK, BTK and TKI inhibitors, has been demonstrated to be an effective method of precisely blocking the transmission of pathological immune signals. Monoclonal antibodies, on the other hand, enable precise intervention at specific immune targets. In addition, cell therapy products based on MSCs and Tregs have opened new therapeutic avenues for refractory cGVHD patients. In light of the expanding array of treatment options, the clinical management of cGVHD is undergoing a transformation towards a dual-track approach that emphasises diversification and personalisation. The focus of future treatment optimisation will be on the rational combination of therapies with different mechanisms and the scientific sequencing of treatment timelines. The objective of this research is to further enhance disease remission depth, improve long-term patient quality of life, and ultimately achieve sustained restoration of immune homeostasis and long-term disease control.

## Data Availability

No datasets were generated or analyzed during the current study.

## Data Availability

No datasets were generated or analyzed during the current study.
